# Arbuscular mycorrhiza suppresses microbial abundance, and particularly that of ammonia oxidizing bacteria, in agricultural soils

**DOI:** 10.3389/fmicb.2025.1671859

**Published:** 2025-11-18

**Authors:** Daquan Sun, Petr Šmilauer, Petra Pjevac, Martin Rozmoš, Sándor T. Forczek, Michala Kotianová, Hana Hršelová, Petra Bukovská, Jan Jansa

**Affiliations:** 1 Laboratory of Fungal Biology, Institute of Microbiology, Czech Academy of Sciences, Praha, Czechia; 2 Faculty of Science, University of South Bohemia in České Budějovice, České Budějovice, Czechia; 3 Joint Microbiome Facility/Division of Microbial Ecology (DOME), Centre for Microbiology and Environmental Systems Science, University of Vienna, Vienna, Austria

**Keywords:** agricultural soils, ammonia oxidizers, arbuscular mycorrhiza, bioassay, compartmented microcosm, environmental gradient, indigenous soil microbiomes, *Rhizophagus irregularis*

## Abstract

Interactions between arbuscular mycorrhizal (AM) fungi and ammonia-oxidizing (AO) microorganisms, two important microbial guilds contributing to soil-plant mineral nutrient cycling, are complex, given the high variability of soil biological, physical, and chemical properties. In addition, AO microorganisms are generally slow growing and require ample time to establish. Their communities are thus difficult to reconstruct under laboratory conditions, for example after soil sterilization. Therefore, in this study, we investigated quantitative and compositional responses of indigenous microorganisms occurring in 50 different field soils (collected from grasslands and arable fields) to actively growing mycelium of the AM fungus *Rhizophagus irregularis*. To this end, we quantified the abundance of various microbial guilds including AO bacteria (AOB), AO archaea (AOA), and comammox *Nitrospira* in pot-incubated soils exposed or not to actively growing AM fungus. Across the variety of soils, we observed systematic suppression by the AM fungus of different microbial groups including bacteria, protists, and fungi. The strongest suppression was noted for AOB and comammox *Nitrospira*, whereas the abundance and community structure of AOA remained unaffected by the AM fungal activity. Mycorrhizal suppression of AOB abundance was accompanied by changes in AOB community structure and correlated with soil pH. Contrary to the expected competition between AM fungus and AO microorganisms for available ammonium (NH_4_^+^) in the soil solution, the presence of the actively growing AM fungus significantly increased soil NH_4_^+^ levels as compared to the non-mycorrhizal control, at least upon the final destructive harvest. Thus, the interaction between the AM fungi and AO microorganisms likely goes beyond the simple competition for the free ammonium ions and might involve microorganisms active in other pathways of soil nitrogen cycle (e.g., mineralization) or temporarily different trajectories of nutrient use in mycorrhizal vs. non-mycorrhizal systems. Alternatively, elusive biological nitrification inhibitors may have contributed to the observed effect, produced by the AM fungus or its host plant, and subsequently transported to the root-free soil via the AM fungal hyphae.

## 1 Introduction

Arbuscular mycorrhizal (AM) fungi form symbiotic relationships with most terrestrial plants, playing a crucial role in the uptake and transfer of nutrients from soil to plants (Lanfranco et al., 2018; Genre et al., 2020). Despite being a relatively small phylogenetic group within the kingdom Fungi, AM fungi from the subphyla *Glomeromycotina* and *Mucoromycotina* significantly impact plant nutrition, particularly the acquisition of phosphorus (P) and nitrogen (N), in addition to micronutrients such as zinc and copper (Bonfante and Venice, 2020). Unlike saprotrophic organisms, AM fungi lack the genetic capacity to synthesize and secrete the necessary exoenzymes to utilize organic nutrients from soil directly (Jansa et al., 2019). Instead, they rely on other microbial guilds, such as bacteria, other fungi, and protists, to decompose (mineralize) organic matter and release nutrients into the soil solution (Bukovská et al., 2018; Rozmoš et al., 2022; Vaishnav et al., 2025). Additionally, AM fungal hyphae can act as conduits or “highways” facilitating the movement of bacteria toward nutrient-rich patches through the production of exudates (Emmett et al., 2021; Jiang et al., 2021; Faghihinia et al., 2024, Vieira et al., 2025).

The uptake of ammonium ions from soil solution and their transfer towards plants by AM fungi is a crucial process for efficient N utilization by plants (Bell et al., 2022). In addition to being a highly favored N source for the AM fungi and their host plants, ammonium is also a substrate for nitrification, a process driven primarily by ammonia-oxidizing (AO) microorganisms, occurring both among bacteria and archaea (Rotthauwe et al., 1997; Leininger et al., 2006; Prosser and Nicol, 2012; Spang et al., 2012; Daims et al., 2015). The microbial conversion of soil-bound ammonia into more leachable nitrate can lead to significant N losses from soil and pollution of surface water bodies and groundwater reservoirs (Cameron et al., 2013). Additionally, ammonium can, through the processes of nitrification and partial denitrification, lead to the production of nitrous oxide (N_2_O), a potent greenhouse gas (Domeignoz-Horta et al., 2018). While nitrification is often limited by the rate of its first step, i.e., oxidation of ammonia to nitrite, conducted by AO prokaryotes, various other processes contribute to full soil nitrification. These include canonical autotrophic nitrite oxidation, heterotrophic nitrification driven by fungi (Martikainen, 2022), and complete nitrification by comammox *Nitrospira* species (Daims et al., 2015). By oxidizing ammonia, AO compete with AM fungi for ammonium ions in soils (Veresoglou et al., 2012; Sun et al., 2024).

It has been shown that the abundance of AO, particularly that of ammonia-oxidizing bacteria (AOB), can be suppressed by the development or activity of AM fungi in artificial substrates (Veresoglou et al., 2019; Dudáš et al., 2022). However, results from studies conducted in natural soils have been rare and inconsistent. For instance, Teutscherova et al. (2019) reported that indigenous AM fungi increased the abundance of AOB following urea application, whereas Wattenburger et al. (2020) found that AM fungi did not affect either AOB or AOA abundances in N-rich soils. In our recent study, AM fungal inoculation suppressed AO abundance in eight agricultural soils (Sun et al., 2024). However, the limited number of agricultural soils examined did not capture the vast diversity of soil properties encountered in agricultural lands and beyond. Furthermore, the presence of potentially highly diverse indigenous AM fungal communities could cause unpredictable effects due to variation in their N uptake and use efficiencies (Delroy et al., 2024). Additionally, AM fungi may stimulate, through priming, i.e., by providing easily available carbon to certain soil saprotrophic microbes, mineralization of soil organic matter and thus the release of ammonium ions from soil organic complexes (ammonification) (Bukovská et al., 2018; Frey, 2019). This could influence the interaction between AM fungi and AO by altering soil ammonium availability.

**FIGURE 1:**
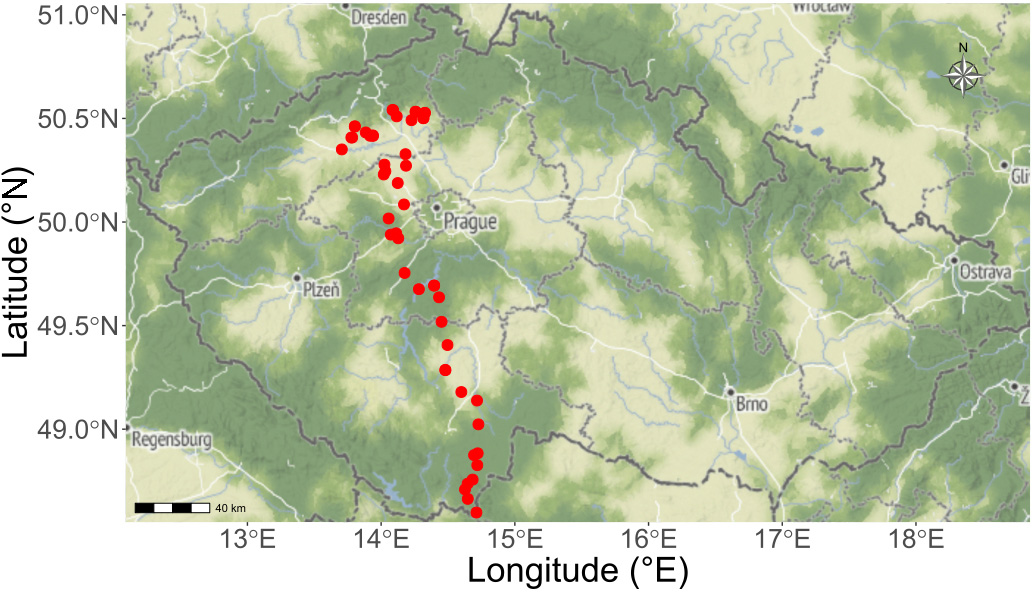
Collection sites of the soils used subsequently in the pot experiment. Soils were obtained from 50 field sites across the Czech Republic (red dots projected on the geographical map) in spring 2023. Soils originated from both grasslands and croplands, covering a variety of soil properties (such as soil textures and nutrient availabilities). The ranges of soil properties are given in Table 1 and full details then in Supplementary Data.

**FIGURE 2:**
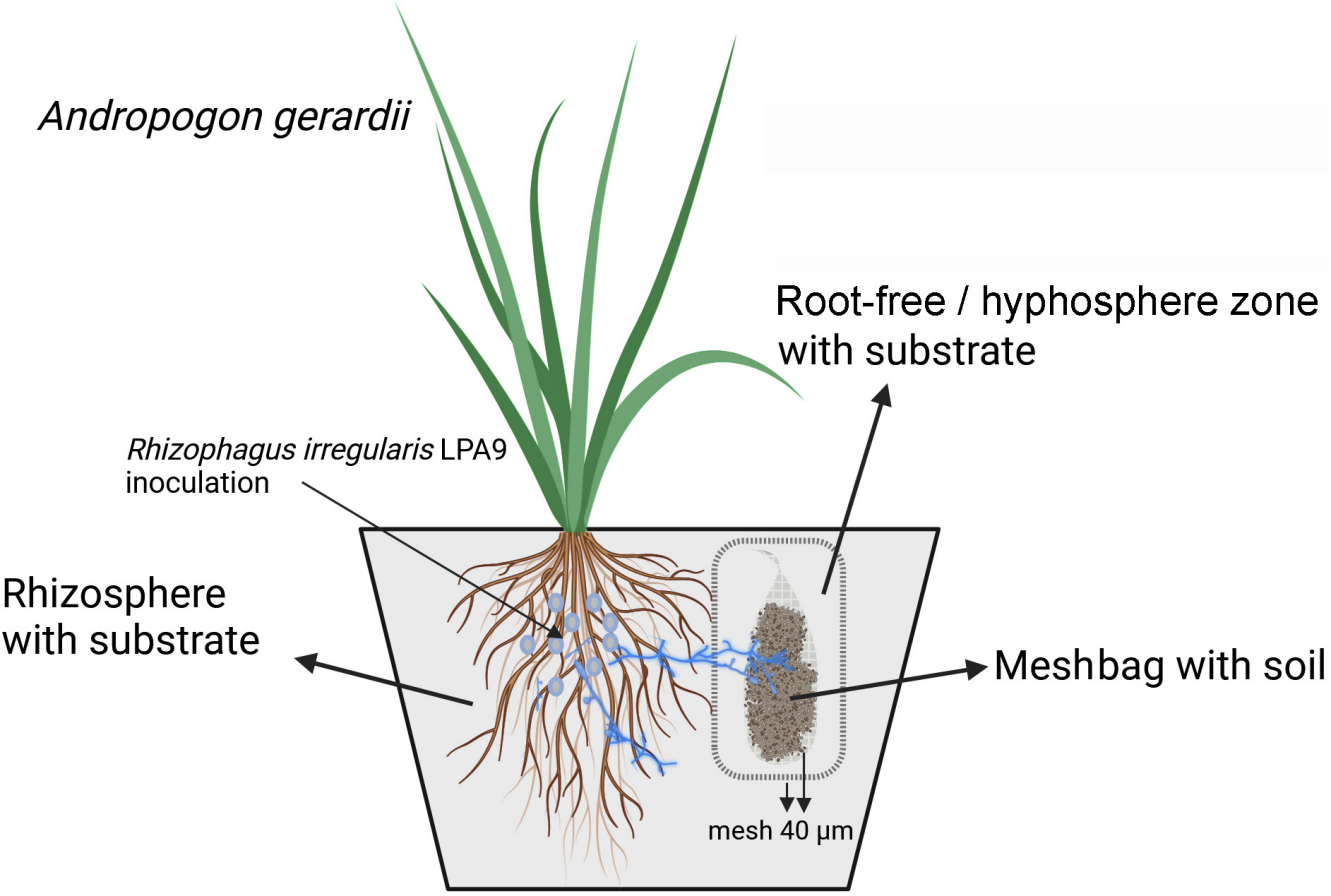
Schematic of the experimental pot setup. Pots had three compartments: (i) rhizosphere filled with a plant cultivation substrate, (ii) root-free zone filled with the same substrate, and (iii) meshbags filled with (unsterile) agricultural soils. Firstly, the arbuscular mycorrhizal (AM) fungus *Rhizophagus irregularis* was inoculated or not in the rhizosphere compartment and allowed to form a symbiosis with the host plant. After 38 days, meshbags (made of 40 μm polyamide mesh fabric) and filled with one of each of the 50 agricultural soils were inserted into pre-installed mesh containers made of polyamide mesh fabric (40 μm). The double 40 μm mesh layers ensured only hyphae, not roots or root hairs, could reach the agricultural soils placed in the meshbags.

To resolve the contribution of the above-mentioned confounding factors and to further explore interactions between AM fungi and other microorganisms (and particularly the AO microorganisms) in a variety of soils, we investigated the responses of indigenous microbial communities to the AM fungus *Rhizophagus irregularis*. Given the high variability in soil biological, physical, and chemical properties and their different effects on AM fungi and/or AO microorganisms (Wattenburger et al., 2020), it appears crucial to study a wide range of soils rather than relying on highly replicated experimental designs using only a limited number of soils, as done in most previous studies (Chen et al., 2013; Dudáš et al., 2022; Sun et al., 2024). We hypothesized that: (1) based on different ecophysiologies, indigenous AOB, comammox *Nitrospira*, and AOA would react differently to AM fungal activity; (2) AM fungi would be more competitive for ammonium than the AO microorganisms and, in consequence, suppress their abundances; and (3) the responses of indigenous AO microorganisms to AM fungal activity would vary with soil properties. Our well-controlled experimental design minimizes root interference, excludes indigenous AM fungi interference in non-mycorrhizal controls by a proper timing of soil addition to pots (see Sun et al., 2024, for more details), and incorporates a diverse array of agricultural soils (Figures 1, 2).

## 2 Materials and methods

### 2.1 Agricultural soil collection

Agricultural soils from 50 field sites (three cores for each site, 10 cm in diameter and 15 cm depth) of different legacies (arable field or grassland) were collected throughout a north-south gradient crossing the Czech Republic between 25th April and 2nd May 2023. These 50 sampling sites range in latitude from 48.49°N to 50.54°N and longitude from 13.70°E to 14.75°E, forming about 200-km long gradient, whereas altitude ranges from 150 m to 940 m above sea level (see Figure 1, Table 1, and Supplementary Data for more details). Stones, large roots and organic debris were removed from the naturally wet soils, and the soils were individually sieved (8 mm mesh) using ethanol-sterilized sieves. Two kg of each such soil (referred to as parent soils in the text below) was stored without any further treatment at 4°C for use in the pot bioassay described below. Physico-chemical properties of the parent soils such as total N and available P concentrations, pH, and biological properties including the abundance and community composition of AOB, AOA, comammox *Nitrospira*, bacteria, fungi and protists are provided in Table 1 and Supplementary Data.

**TABLE 1:** Range of elevations and selected (parent) soil properties across sampling sites.

Parameter	Minimum	Median	Maximum
Altitude (m above sea level)	150	360	940
pH (water)	5.14	7.33	8.11
NH_4_^+^ (μg/g)	0.64	1.54	36.6
NO_2_^–^ (μg/g)	0.03	0.12	0.83
NO_3_^–^ (μg/g)	0.96	11.3	66.9
Total mineral N (μg/g)	2.85	12.9	93.5
Water extractable phosphorus (μg/g)	0.06	3.63	58.1
Sand (%)	8.90	38.6	78.7
Silt (%)	10.4	31.9	62.7
Clay (%)	5.57	28.2	66.7
Total N (%)	0.10	0.19	0.51
Total organic C (%)	1.01	2.24	7.83
AOB *amoA* (gene copies/mg soil)	71.8	8423	63416
AOA *amoA* (gene copies/mg soil)	1879	271014	1606999

Most of the explorative data analyses were conducted using 50 fields soils before incubation in the pots, except NO_2_^–^ concentration (μg/g) which could only have been conducted on 32 soils since 18 samples were under detection limit. Analyses of mineral nitrogen (N) species were conducted spectrophotometrically using 2 M KCl extracts. Total mineral N is a sum of concentrations of ammonium, nitrite and nitrate ions. Total N and total organic carbon (C) analyses were conducted by combustion elemental analyzer. Concentrations of *amoA* gene of ammonia oxidizing bacteria (AOB) and ammonia oxidizing archaea (AOA) were measured by quantitative real-time PCR. See Supplementary methods for more details.

### 2.2 Experimental setup and design

A compartmented pot experiment was conducted focusing on the interaction between actively growing mycelium of the AM fungus *Rhizophagus irregularis* and indigenous microbial communities present in the different soils (Figure 2). The design featured meshbags made of fine nylon mesh that only allowed penetration of AM fungal hyphae but not roots (Dudáš et al., 2022; Sun et al., 2024). In addition, through delayed administration of the soil-containing meshbags to the pots, the probability of colonization of the host plant by indigenous AM fungi from the parent soils was reduced, which was particularly important for keeping the non-mycorrhizal control plants free of mycorrhizal colonization.

Specifically, the pots (20 cm × 11 cm × 11 cm, h × l × w) contained 2 L of cultivation substrate; a mixture (45:45:10, v:v:v) of autoclaved quartz sand, autoclaved zeolite (2.5–4 mm grain size), and γ-irradiated (25 kGy) soil from Litoměřice, Czech Republic, supplied with microbial suspensions from previous (3.5 years) non-mycorrhizal pot cultures planted in leeks (Řezáčová et al., 2016). Physico-chemical properties of the substrate have been described previously (Dudáš et al., 2022). Plants (*Andropogon gerardii*), either inoculated or not with *in vitro* produced hyphae and spores of *Rhizophagus irregularis* LPA9 (Rozmoš et al., 2022), were growing outside the 500-mL plastic cylinders with permeable walls and bottom (2-mm openings, cat. no. P00718, Annelli, Montanaso Lombardo, Italy) covered with a nylon mesh fabric (40-μm mesh openings; commercially available as Uhelon 130T; Silk & Progress, Brněnec, Czech Republic). The edge of the cylinders was at the height of the rim of the pots, i.e., slightly higher than the substrate level. Empty centrifugation vials (50 mL) were placed in the middle of the plastic cylinders as placeholders for soil containing meshbags to be applied at a later timepoint.

Fifty seeds of *Andropogon gerardii* (Jelitto Staudensamen, Schwarmstedt, Germany) were sown 1 cm below the substrate surface of each pot and inoculated with approximately 30,000 AM fungal spores and uncounted hyphal fragments per pot, 2 cm below the surface in the rhizosphere compartment of each mycorrhizal pot. Non-mycorrhizal control pots were established similarly, only omitting the input of living AM fungal inoculum. Fifty mycorrhizal and fifty non-mycorrhizal pots were established. A small amount (up to 2 mL daily) of deionized water was added to the surface of each pot using a water nebulizer twice a day until plants germinated. After plant emergence (approximately 3 days after sowing), deionized water was applied once a day to maintain gravimetric water content between 18 and 24% (corresponding to 60 and 80% of the water holding capacity of the substrate, respectively).

At day 38 after sowing, two aliquots of each of the 50 living parent soils (30 g each) were added to individual meshbags (made of 40 μm nylon fabric as specified above), sealed with zip-ties and placed inside the cylinders, replacing the empty centrifugation vials, one into a mycorrhizal, and one into a non-mycorrhizal pot. Positions of the 100 pots were completely randomized in the glasshouse and pot positions were further rotated (i.e., swapped mirror-wise) twice during the subsequent incubation (35 days) to mitigate systematic effects of microclimatic condition variations in the glasshouse. Throughout the experiment (lasting 73 days altogether), the pots were incubated in the glasshouse of the Institute of Microbiology in Prague, Czech Republic, with controlled temperatures and light supplemented with high-pressure metal-halide lamps, providing a minimum of 240 μmol photosynthetically active radiation m^–2^ s^–1^ during a 16 h photoperiod. Light and temperature records from the glasshouse are available in Supplementary Data.

Starting at 5th week after sowing, 65 mL of Long Ashton nutrient solution (Hewitt, 1952, also see the composition of the nutrient solution in the Supplementary Data) with reduced P concentration (containing 0.52 mg P in the form of orthophosphate) and full concentration of nitrate (10.9 mg N) was added to the plant compartment of each pot. This continued every week, supplying in total of 3.12 mg P and 65.4 mg N (almost exclusively in nitrate form) per pot, besides other nutrients.

### 2.3 Harvest and measurements

Plant biomass and soil samples from the meshbags were collected from all 100 pots at day 73 after sowing. Plant roots (after removing a fresh representative root sample for staining and mycorrhizal colonization determination, with fresh weights recorded before and after removal of the portion for staining) and shoots were dried at 65°C for 3 days, and their dry weights per pot were determined. Representative samples of the soils were collected from the meshbags (approx. 20 g fresh weight per sample) and dried at 65 °C for 3 days. Both plant and soil samples were then pulverized using a MM200 ball mill (Retsch, Haan, Germany) at 25 Hz for 2 min, employing two stainless steel balls (10 mm diameter) per sample. Total P content in plant shoots and roots (using subsamples of 0.1 g each) was determined by the malachite green method (Ohno and Zibilske, 1991) following incineration and concentrated HNO_3_ extraction as described previously (Püschel et al., 2017). Total N and total organic C in the plant biomass (2 mg) and soils (20 mg) were analyzed using the Flash EA 2000 elemental analyzer (Thermo Fisher Scientific, Bremen, Germany). Soil pH was measured in 1:2.5 (w:v) aqueous suspensions. Soil texture was analyzed using H_2_O_2_ treated soil samples as described previously (Řezáčová et al., 2019).

Ammonium concentration in soil was quantified in soil KCl (2 M, 1:5 fresh weight:volume) extracts by a modified indophenol method based on the classical Berthelot reaction (Hood-Nowotny et al., 2010). Nitrite and nitrate were determined by the Griess method, first quantifying the concentration of nitrite alone, then converting nitrate to nitrite and quantifying the sum of both (Hood-Nowotny et al., 2010). Total mineral N in soil was calculated as a sum of N represented by each of the three mineral N species above, while considering the ratio between dry and fresh weights of the soil.

Roots were stained with Trypan blue (Koske and Gemma, 1989), and colonization was assessed microscopically by observing 50 root intersections per sample under a compound microscope (magnification 200×; McGonigle et al., 1990).

### 2.4 DNA extraction and qPCR

Soils (∼250 mg dry powder) from the fields (i.e., parent soils) and from the meshbags were used for DNA extraction using the DNeasy PowerLyzer kit (Qiagen, Venlo, Netherlands). An internal DNA standard containing 2 × 10^10^ gene copies of cassava mosaic virus (Thonar et al., 2012) was added to each sample prior to DNA extraction to determine DNA extraction efficiency. Specific primers (with or without a TaqMan probe) were used to measure abundances separately for the inoculant AM fungus (*R. irregularis*), bacteria, non-mycorrhizal fungi, and protists, and the abundance of ammonium monooxygenase (*amoA*) gene for AOB and AOA, as well as the comammox *Nitrospira*, as detailed previously (Dudáš et al., 2022; Blom et al., 2024; Supplementary methods). Dissimilatory nitrate reduction to ammonium (DNRA) gene primers targeting the *napA* (Bru et al., 2007) and *nrfA* (Cannon et al., 2019) genes were used to quantify the abundance of microorganisms carrying these genes in the different soils. Quantitative real-time PCR (qPCR) was conducted using the LightCycler 480 II instrument (Roche, Rotkreuz, Switzerland) as specified in Supplementary methods. For calibration, dilution series were prepared from the relevant amplicons as described previously (Thonar et al., 2012; Dudáš et al., 2022). The qPCR quantification was carried out in 96-well plates using a 20-μL final reaction volume (more of technical details are available in Supplementary methods). Either Luna universal probe qPCR master mix (M3004; for assays including a TaqMan probe) or Luna universal qPCR master mix (M3003; containing SYBR green, for assays without a probe) were used, both supplied by New England Biolabs (Ipswich, MA, United States). Fluorescence was recorded employing the SYBR green/fluorescein color channel. Results of the qPCR analyses were corrected for internal DNA standard recoveries and exact soil sample weights.

### 2.5 Microbial community analyses

The communities of soil prokaryotes (16S rRNA gene), protists (18S rRNA gene), AOA (*amoA* gene) and AOB (*amoA* gene) were analyzed both in the parent soils and in the soil samples recovered from the meshbags, using massively parallel amplicon sequencing on the Illumina MiSeq (v3, 600 cycles, 2 × 300 bp) platform, employing the primers and procedures described previously (Dudáš et al., 2022). For the parent soils, we also analyzed the composition of the indigenous AM fungal communities as detailed previously (Bukovská et al., 2021). For technical details of PCR steps constituting the amplicon preparation, please also see Supplementary methods.

MiSeq paired-read sequencing data were merged (overlap = 20), quality filtered (Phred quality scores per sequence ≥ 30, Phred quality score per base ≥ 7), potential chimeras removed, and sequences clustered at 97% similarity level (resulting in operational taxonomic units, OTUs), using the SEED 2.0 software (Větrovskỳ et al., 2018) and parameters described previously (Dudáš et al., 2022), and preliminarily identified using following databases: NCBI^1^ for AOA and AOB, SILVA^2^ for prokaryotes and AM fungi, and PR2^3^ for protists. Contaminating sequences such as mitochondrial and chloroplast sequences within the prokaryotic 16S rRNA gene dataset, or fungal sequences within the protistan 18S rRNA gene dataset were removed, and sequencing outputs then rarefied to 15,700 sequences per sample (prokaryotes), 6,000 sequences per sample (protists), 12,000 sequences per sample (AOB), 9950 sequences per sample (AOA) and 2,000 sequences per samples (AM fungi). The rarefied data were again subjected to potential chimera search and removal and sequences were subsequently clustered at 97% similarity level, representative sequences of each OTU identified by using the databases quoted above, and the OTUs merged at GenBank accession level (AOB and AOA) or genus level (prokaryotes and protists, and AM fungi), wherever possible. In case the genus was not identifiable for a specific OTU, the next higher taxon (with prefix “un_”) was used. Altogether, 644 taxa were included in the subsequent multivariate analyses for AOB, 388 taxa for AOA, 741 taxa for prokaryotes and 1,104 taxa for protists (see Supplementary Data for details). The sequences were deposited in the Sequence Read Archive of the NCBI under the accession number PRJNA1085112.

### 2.6 Statistical analyses

The mycorrhizal response ratio (MRR) was calculated to quantify the AM fungal inoculation effect by comparing paired values obtained from mycorrhizal and non-mycorrhizal pots added with the same soil as follows:


$$M\,R\,R=l\,n\,\frac{V\,a\,l\,u\,e\,f\,r\,o\,m\,a\,m\,y\,c\,o\,r\,r\,h\,i\,z\,a\,l\,p\,o\,t\,a\,d\,d\,e\,d\,w\,i\,t\,h\,s\,o\,i\,l+1}{V\,a\,l\,u\,e\,f\,r\,o\,m\,a\,n\,o\,n-m\,y\,c\,o\,r\,r\,h\,i\,z\,a\,l\,p\,o\,t\,a\,d\,d\,e\,d\,w\,i\,t\,h\,s\,o\,i\,l+1}$$

The MRR < 0 implies suppression due to AM fungal inoculation, MRR > 0 implies promotion due to AM fungal inoculation, and MRR = 0 implies no effect of the AM fungal inoculation on the specific variable.

Fulfilling assumptions of ANOVA were checked for the different data (either untransformed or the MRR values) by Shapiro-Wilk test for normality, and Levene’s test for homogeneity of variances using the “car” package in R 4.2.2 (R Core Team, 2022). Since the ANOVA assumptions were usually not met, we used the one-sample Wilcoxon signed-rank test to test whether the medians of MRR for a specific variable were significantly different from a hypothesized value (mu = 0) or the two-sample Wilcoxon signed-rank test to determine differences between mycorrhizal and non-mycorrhizal treatments. Further, the MRR of AOA, AOB, and comammox *Nitrospira* (all targeting the *amoA* gene abundances) in the meshbags were correlated with the parent soil properties and MRR of DNRA gene abundances, employing the “Spearman” approach.

Principal component analysis (PCA) and redundancy analysis (RDA) (Legendre and Legendre, 2012) were used, respectively, to analyze the sources of variation in microbial community datasets or to quantify and test the community variation explained by particular predictor(s). Contour plots were constructed based on local polynomial regression models (loess, Cleveland, 1979) fitted to values of Hill’s N2 diversity index (Legendre and Legendre, 2012) with the case positions on two PCA axes used as predictors. To test the effect of incubation of soils in the pots, we performed partial RDA with soil identity (factor with 50 levels) used as a covariate and permuted the three sample types within each soil identity, while for testing the effect of AM fungal hyphae presence, we again used the soil identity as a covariate and permuted the two samples coming from the two pots with or without the presence of *R. irregularis*. To identify microbial taxa responding to a particular predictor type, we used *t*-value biplots as described previously (ter Braak and Šmilauer, 2018, section 5.7). Multivariate analyses were all conducted in Canoco 5.15 software (ter Braak and Šmilauer, 2018).

## 3 Results

**FIGURE 3:**
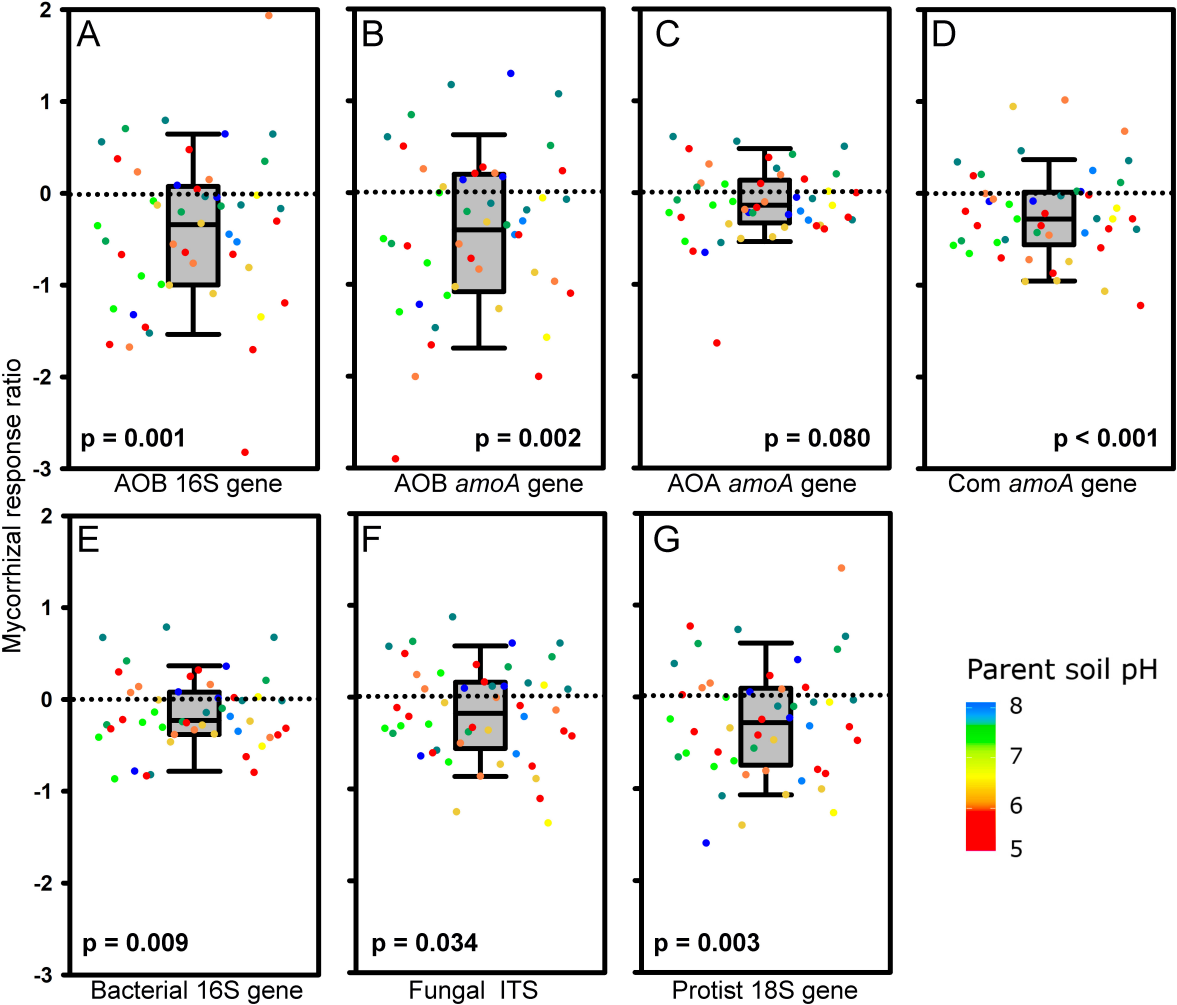
Mycorrhizal response ratio (MRR; *n* = 48, excluding two soils, IDs 13 and 15, where indigenous arbuscular mycorrhizal fungi reached out from the meshbags and formed symbiosis with originally non-mycorrhizal plants) of abundances of **(A)** 16S rRNA gene of ammonia oxidizing bacteria (AOB), **(B)** ammonia monooxygenase (*amoA*) gene of AOB, **(C)** *amoA* gene of ammonia oxidizing archaea (AOA), **(D)** *amoA* gene of comammox *Nitrospira*, **(E)** 16S rRNA gene of bacteria, **(F)** internal transcribed spacer (ITS) of fungi, and **(G)** 18S rRNA gene of protists. MRRs are displayed as medians, 25 and 75% percentiles (boxes) and 5 and 95% percentiles (error bars) of MRRs calculated separately for each soil (dots), using individual values measured in meshbags placed in mycorrhizal (AM) and non-mycorrhizal (NM) pots. The *p*-values refer to nonparametric one-sample Wilcoxon signed-rank test testing differences of sample median from zero. Dot colors stand for pH values of the parent soils.

Presence of living AM fungal hyphae consistently and strongly suppressed AOB and comammox *Nitrospira* abundance across the different soils, as detected by qPCR targeting either the 16S rRNA gene in the AOB or the *amoA* genes of the respective microorganisms (Figures 3A, B, D). In contrast, the abundance of AOA was not significantly affected by the presence of AM fungal hyphae (Figure 3C). Other microbial guilds including bacteria, non-mycorrhizal fungi, and protists were also suppressed by the actively growing AM fungus (Figures 3E–G), although the extent of suppression was somewhat lower than the suppression observed for the AOB and comammox *Nitrospira* (Figure 3). High variability between individual soils often precluded the suppression effect from being statistically significant in unpaired comparisons (Table 2), in contrast to pairwise comparisons presented in Figure 3.

**TABLE 2:** Differences in plant/microbial parameter measured in the pots (either on plants or in the meshbags) as affected by inoculation of the plants with arbuscular mycorrhizal fungus *Rhizophagus irregularis* LPA9 (AM) vs. non-mycorrhizal treatment (NM) across the different soils filled in the meshbags, as per the non-parametric Wilcoxon signed-ranked test.

Compartment	Parameter	NM	AM	*p*-value
Plant	Shoot biomass (g)	1.37	3.52	**<0.001**
Plant	Root biomass (g)	1.63	5.26	**<0.001**
Plant	Total biomass (g)	2.96	8.84	**<0.001**
Plant	Root nitrogen (N) concentration (%)	1.08	0.71	**<0.001**
Plant	Shoot N concentration (%)	1.34	1.05	**<0.001**
Plant	Shoot N content (mg)	18.3	35.8	**<0.001**
Plant	Root N content (mg)	17.1	37. 5	**<0.001**
Plant	Plant N content (mg)	36.6	72.5	**<0.001**
Plant	Root phosphorus (P) concentration (mg/g)	0.69	1.25	**<0.001**
Plant	Shoot P concentration (mg/g)	0.48	1.12	**<0.001**
Plant	Root P content (mg)	1.15	6.63	**<0.001**
Plant	Shoot P content (mg)	0.65	3.76	**<0.001**
Plant	Plant P content (mg)	1.82	10.5	**<0.001**
Plant	Rhizophagus in roots	909	2726561	**<0.001**
Meshbag	NO_3_^–^ concentration (μg/g)	15.3	1.03	**<0.001**
Meshbag	NO_2_^–^ concentration (μg/g)	0.205	0.227	0.416
Meshbag	NH_4_^+^ concentration (μg/g)	0.902	1.374	0.097
Meshbag	Total N concentration (%)	0.187	0.197	0.592
Meshbag	Total organic C concentration (%)	2.012	2.012	0.927
Meshbag	AOB 16S rRNA gene (copies/mg soil)	1323	1031	0.099
Meshbag	Bacterial 16S rRNA gene (copies/mg soil)	664104	573545	**0.043**
Meshbag	Fungal ITS (copies/mg soil)	30200	22425	0.116
Meshbag	Rhizophagus in soil	0	19217	**0.000**
Meshbag	Protist 18S rRNA gene (copies/mg soil)	408886	277776	**0.021**
Meshbag	AOB *amoA* gene (copies/mg soil)	7445	5764	0.138
Meshbag	AOA *amoA* gene (copies/mg soil)	195613	167996	0.779
Meshbag	Comammox *amoA* gene (copies/mg soil)	6577	5715	**0.026**

Median values (n = 48) are shown. Rhizophagus: *Rhizophagus irregularis* abundance measured by quantitative real-time PCR through targeting mitochondrial large ribosomal subunit (LSU) gene (mt5 marker, copy number/mg roots or soil); AOB *amoA*: *amoA* gene abundance of ammonia oxidizing bacteria (copy number/mg soil); AOA *amoA*: *amoA* gene abundance of ammonia oxidizing archaea (copy number/mg soil). Statistically significant (*p* < 0.05) differences between the NM and AM treatments are shown in bold. AOB: ammonia oxidizing bacteria; AOA: ammonia oxidizing archaea.

**FIGURE 4:**
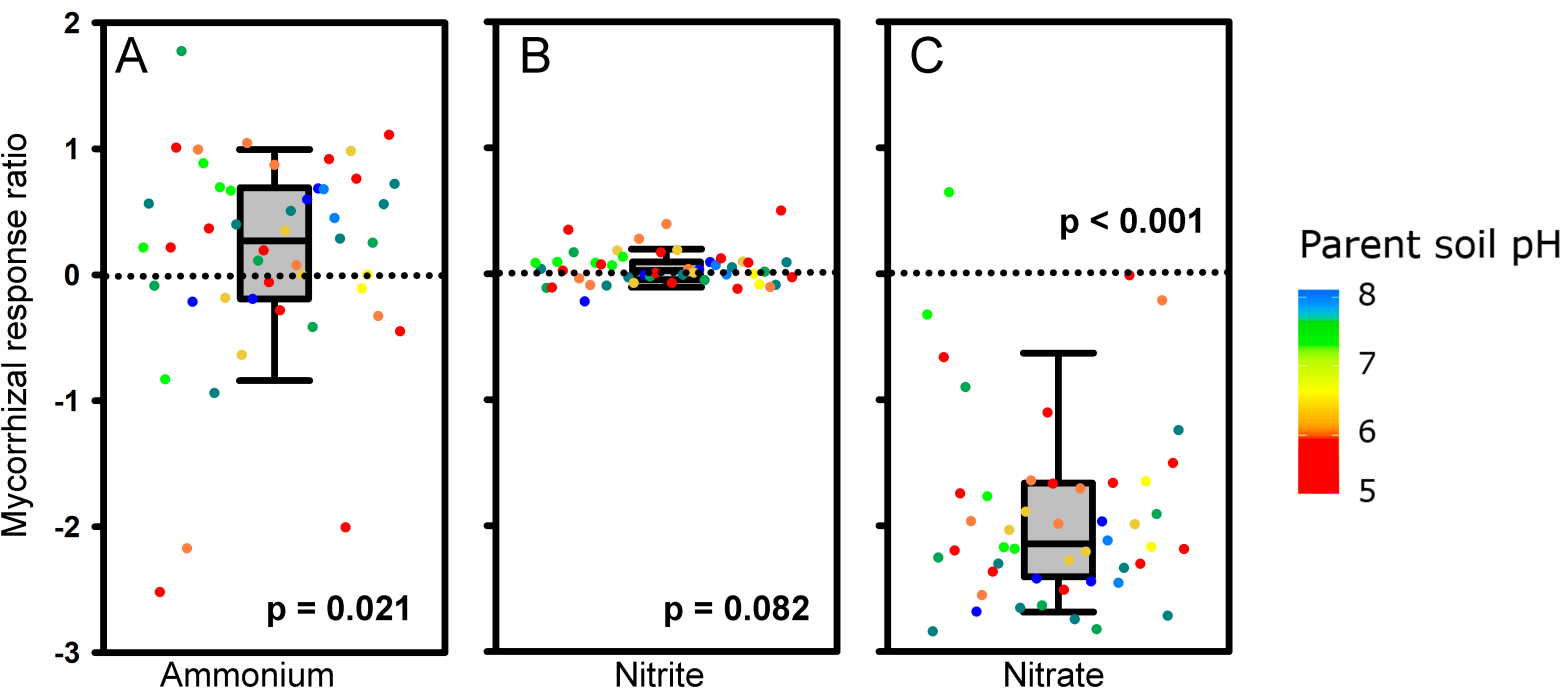
Mycorrhizal response ratio (MRR; *n* = 48, see legend to Figure 3 for more details) of ammonium **(A)**, nitrite **(B)** and nitrate **(C)** concentrations in the meshbags to the inoculation with *Rhizophagus irregularis*. MRRs are displayed as medians, 25 and 75% percentiles (boxes) and 5 and 95% percentiles (error bars) of MRRs calculated separately for each soil (dots), using individual values measured in meshbags placed in mycorrhizal (AM) and non-mycorrhizal (NM) pots. The *p*-values refer to nonparametric one-sample Wilcoxon signed-rank test testing differences of sample median from zero. Dot colors stand for pH values of the parent soils.

Most plant parameters, including biomass, plant P nutrition (both the concentrations and contents) and N content (but not N concentrations in either shoots or roots), were positively affected by mycorrhizal inoculation (Tables 2, 3). Particularly interesting was the effect of AM inoculation on the ammonium, nitrite, and nitrate concentrations in the meshbags. When analyzed as relative mycorrhizal responses, i.e., accounting for natural variability of these properties across the different soils, ammonium concentration was generally higher, whereas nitrate concentration was lower in mycorrhizal as compared to non-mycorrhizal meshbags, and the nitrite concentration was not affected by the presence of actively growing AM fungus (Figure 4 and Table 3). The positive effect of AM fungal inoculation on ammonium concentration in the meshbags vanished upon unpaired comparison, whereas the negative effect on nitrate concentration was retained (Table 2). The abundance of organisms carrying the DNRA genes (*napA* or *nrfA*) was in general significantly suppressed (*p* < 0.001) by AM fungal inoculation (Supplementary Figure 1).

**TABLE 3:** Relative responses to inoculation of the plants with arbuscular mycorrhizal (AM) fungus *Rhizophagus irregularis* LPA9 with respect to plant parameters and mycorrhizal colonization of the roots and meshbags (filled with the different soils, *n* = 48) recovered from the mycorrhizal and non-mycorrhizal pots.

Compartment	Parameter	Median	*p*-value
Plant	Shoot biomass (g)	0.284	**<0.001**
Plant	Root biomass (g)	0.396	**<0.001**
Plant	Total biomass (g)	0.407	**<0.001**
Plant	Root nitrogen (N) concentration (%)	-0.069	**<0.001**
Plant	Shoot N concentration (%)	−0.058	**<0.001**
Plant	Shoot N content (mg)	0.268	**<0.001**
Plant	Root N content (mg)	0.332	**<0.001**
Plant	Plant N content (mg)	0.306	**<0.001**
Plant	Root phosphorus (P) concentration (mg/g)	0.110	**<0.001**
Plant	Shoot P concentration (mg/g)	0.146	**<0.001**
Plant	Root P content (mg)	0.548	**<0.001**
Plant	Shoot P content (mg)	0.461	**<0.001**
Plant	Plant P content (mg)	0.603	**<0.001**
Plant	Rhizophagus in roots	3.493	**<0.001**
Meshbag	NO_3_^–^ concentration (μg/g)	−0.943	**<0.001**
Meshbag	NO_2_^–^ concentration (μg/g)	0.008	0.089
Meshbag	NH_4_^+^ concentration (μg/g)	0.117	**0.022**
Meshbag	Total N concentration (%)	0.003	**0.002**
Meshbag	Total organic C concentration (%)	0.000	0.750

One-sample Wilcoxon signed-rank test results are provided, scrutinizing whether medians of the different parameters significantly differed from zero. Positive and negative median values indicate promotion and suppression, respectively, in the AM inoculated vs. non-mycorrhizal treatments. For explanation of the different parameters please see legend to Table 2. Statistically significant (*p* < 0.05) differences of medians from zero shown in bold.

The MRR of AOB abundance was significantly and positively correlated with parent soil pH and latitude, as well as the abundance of AOA in the parent soils (Table 4). The MRR of AOA abundance was significantly and positively correlated with total organic C and ammonium concentrations in the parent soils and negatively correlated with soil sandiness (Table 4). The MRR of comammox *Nitrospira* was significantly and positively correlated with latitude, soil clay content, total organic C content, total N, and available ammonium concentrations. Further, it correlated negatively with soil sandiness (Table 4). The MRRs of all the three above groups of AO microorganisms were significantly and positively correlated with MRRs of the two genes involved in the DNRA pathway (Table 4).

**TABLE 4:** Correlations matrix (Spearman) between mycorrhizal response ratios (MRRs) of abundance of ammonia oxidizing bacteria (AOB), ammonia oxidizing archaea (AOA), and Comammox *Nitrospira* (Com) in the meshbags (*n* = 48) and the parent soil physico-chemical and biological properties, and responses of genes of the dissimilatory nitrate reduction to ammonia (DNRA) pathway (nitrate reductase, *napA*, and nitrite reductase, *nrfA*) to the presence of the AM fungus in the meshbags.

Explanatory variable (parent soil properties or MRR of DNRA genes)	AOB	AOA	Com
pH	**0.341**	0.095	0.214
Latitude	**0.399**	0.278	**0.321**
Altitude	−0.256	−0.026	−0.276
Total mineral nitrogen (N) concentration (%)	0.129	−0.091	0.057
Available phosphorus concentration (μg/g)	−0.132	−0.139	−0.226
Sand (%)	−0.211	−**0.293**	−**0.285**
Silt (%)	0.046	0.115	0.056
Clay (%)	0.166	0.235	**0.314**
Total organic carbon concentration (%)	0.191	**0.394**	**0.473**
Total N concentration (%)	−0.047	0.283	**0.326**
NH_4_^+^ concentration (μg/g)	0.200	**0.318**	**0.288**
NO_3_- concentration (μg/g)	0.126	−0.139	−0.011
AOB (16S rRNA gene copies per mg soil)	0.187	−0.080	−0.145
Bacteria (16S rRNA gene copies per mg soil)	−0.124	0.097	−0.031
Fungi (ITS copies per mg soil)	0.078	0.207	0.217
Rhizophagus (mitochondrial large ribosomal subunit copies per mg soil)	−0.042	0.041	0.029
Protists (18S rRNA copies per mg soil)	0.066	0.213	0.090
AOB *amoA* gene (copies per mg soil)	0.181	−0.078	−0.137
AOA *amoA* gene (copies per mg soil)	**0.370**	−0.028	−0.185
Com *amoA* gene (copies per mg soil)	−0.204	−0.053	−0.205
*napA* gene abundance (copies per mg soil) – MRR in the pot experiment	**0.630**	**0.729**	**0.664**
*nrfA* gene abundance (copies per mg soil)—MRR in the pot experiment	**0.629**	**0.730**	**0.661**

Correlation coefficients are shown and statistically significant correlations (*p* < 0.05) are in bold.

Community composition of the various microbial groups was driven mainly by altitude of the sampling sites and soil sandiness and (in the opposite direction) by soil pH (Supplementary Figure 2). Prokaryotic diversity increased with higher soil available P and lower total N concentrations in the soils (Supplementary Figure 2). Similar drivers were observed for the protistan and AOB communities, whereas the effects of soil physico-chemical parameters on AOA diversity were less clear (Supplementary Figure 2).

**FIGURE 5:**
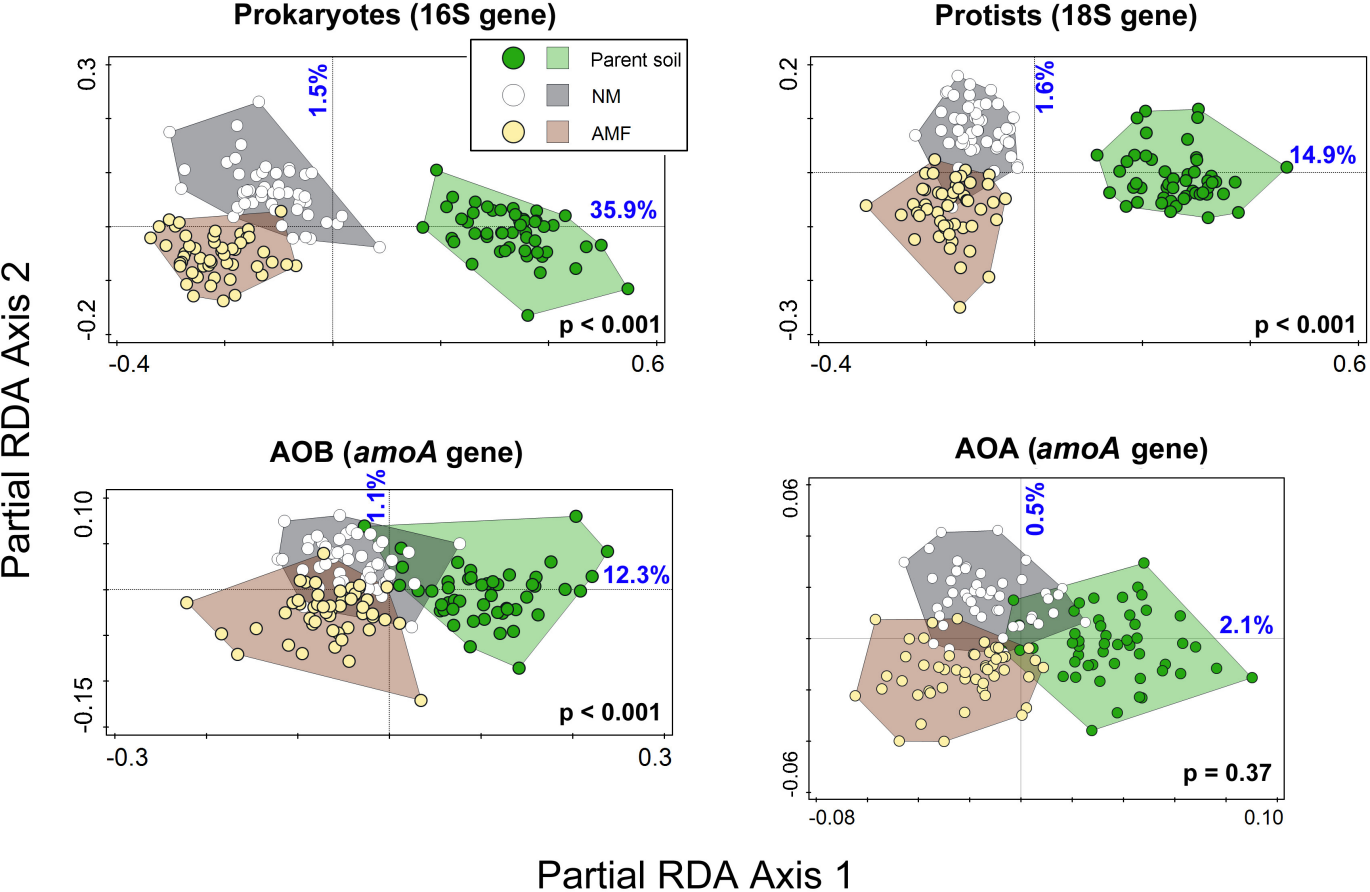
Ordination diagrams from partial redundancy analyses (RDA) using microbial community profiles from field (parent) soils (i.e., soils prior to incubation in the meshbags, green dots and area) and soils incubated in meshbags either in the mycorrhizal (AMF, yellow dots and brown area) or non-mycorrhizal (NM, white dots in gray area) setups. Soil IDs were used as co-variates, restricting permutations. The *p*-values from the permutation test addressing the effect on all axes are shown in bold black writing. Percentage of dataset variability explained by the first and second axes are shown in blue. AOB, ammonia oxidizing bacteria; AOA, ammonia oxidizing archaea.

Detailed microbial community analyses revealed a strong relative community shift due to incubation of the different soils in the meshbags for both prokaryotes and protists and somewhat less strong, albeit still significant effect was recorded for the AOB and AOA communities (Figure 5 and Table 5). The effect of the presence of actively growing AM fungal mycelium in the meshbags was significant for prokaryotes, protists and AOB, but not statistically significant for the AOA community composition (Figure 5 and Table 5).

**TABLE 5:** Fraction of variability of microbial communities as assessed by amplicon sequencing in the field (parent) soils before incubation in the pots, and the same soils incubated in mycorrhizal (AM) or non-mycorrhizal pots, explained either by the effect of cultivation under glasshouse conditions (irrespective in which kind of pots) or the variability due to AM fungal inoculation in the pot cultures.

	1st axis explanatory limit	Effect of glasshouse cultivation (field vs. pot samples)		Effect of AM fungal inoculation	
Microbial community		*R*^2^_adj_ [%]	pseudo-F/*p*-value	*R*^2^_adj_ [%]	Pseudo-F/*p*-value
Prokaryotes (16S)	6.11	5.43	53.0/**<0.001**	0.49	4.7/**<0.001**
Protists (18S)	3.66	3.32	17.0/**<0.001**	0.36	2.4/**<0.001**
AOB (*amoA* gene)	0.79	0.54	13.5/**<0.001**	0.04	1.8/**< 0.001**
AOA (*amoA* gene)	1.04	0.03	2.1/**0.010**	0.00	0.4/n.s.

n.s. not statistically significant. The *1st axis explanatory limit* column was estimated as the variation explained by the first axis of PCA. It represents the maximum amount of variation that can be explained by a predictor with single degree of freedom and therefore establishing a practical limit for values found in the two R^2^_adj_ columns. AM, arbuscular mycorrhizal; AOB, ammonia oxidizing bacteria; AOA, ammonia oxidizing archaea. The *p*-values below 0.05 are shown in bold.

Upon searching for microbial taxa particularly affected in their relative abundance by soil incubation in the meshbags, or the presence of actively growing AM fungus, there were always more taxa lost than gained upon soil incubation in the meshbags for the prokaryotes, protists, and AOB. In contrast, several AOA taxa increased rather than decreased in their relative abundance upon soil incubation in the meshbags (Supplementary Figure 3). Moreover, several taxa within the prokaryotes, protists, and AOB were specifically reacting by increasing or decreasing their relative abundance to the presence of actively growing AM fungal hyphae, but no such taxon could have been identified for AOA (Supplementary Figure 3).

## 4 Discussion

In this study, we experimented with 50 agricultural soils from central Europe representing a wide range of physico-chemical properties to confirm and further deepen our understanding of interactions between AM fungi and AO microorganisms in soil. The delayed insertion of meshbags filled with unsterile agricultural soils into mycorrhizal and non-mycorrhizal microcosms allowed us to exclude interference from indigenous AM fungi (whose composition varied across the different soils, see Supplementary Data for details) that could also form symbiosis with plants. In fact, roots of experimental plants in 48 out of 50 of the non-mycorrhizal pots remained free of mycorrhiza in the experiment reported here, which is why the subsequent analyses were then carried out on only 48 pairs of microcosms, where the absence of AM fungi in the non-mycorrhizal plants was confirmed.

Delayed insertion of the meshbags thus resolved the confounding factor of indigenous AM fungi influencing results, which was the case for our previous study where the meshbags with soil were deposited in the pots right from the beginning of plant cultivation (Sun et al., 2024). The improved experiment setup, compared to previous research, allowed us to confirm the first hypothesis that AM fungi generally suppressed AOB but not the AOA abundances across a wide gradient of soil physico-chemical properties. Besides, we also demonstrated that similar suppression to that recorded for AOB also applied to comammox *Nitrospira*, which has never been specifically tested before, to the best of our knowledge. An interesting observation here was that virtually all microbes except the AOA were suppressed in abundance due to actively growing AM fungal mycelium (Figure 3). In our previous studies, we showed that the AM fungal hyphae rapidly acquired NH_4_^+^ and retained the N or transferred it to plant roots (Rozmoš et al., 2022; Sun et al., 2024; Vaishnav et al., 2025). We hypothesized that the high competitiveness of AM hyphae decreased soil NH_4_^+^ levels and thus reduced the abundance of AO microorganisms. To our surprise, this second hypothesis was not unequivocally confirmed here, as in general AM fungal presence significantly increased NH_4_^+^ concentrations in the meshbag soils, at least at the very end (i.e., upon destructive harvest) of the experiment. This “ammonium paradox” has probably been the most prominent, unexpected, and potentially confusing result of the research presented here, thus deserving dedicated discussion about possible underlying mechanisms.

First, we speculated that the presence of AM fungus might have enhanced the decomposition of organic N compounds in the agricultural soils included in this study, thus replenishing the soil NH_4_^+^ pool. There is evidence showing that AM fungi can acquire NH_4_^+^ from soil organic matter or soil organic amendments via stimulating decomposition/priming of such resources (Frey, 2019; Jansa et al., 2019; Bukovská et al., 2021). However, we did not find increased overall bacterial or fungal abundances after AM fungal inoculation (Table 2 and Figure 3), which somewhat contradicts this explanation of the “ammonium paradox.” Yet, we could not further elaborate on this, as no isotopic tracers were used here.

Second, we thought that the presence of AM fungus might have induced activity of the DNRA pathway in the soils as reported recently (Zhang et al., 2020; Zhao et al., 2025). In this pathway, nitrate is used as an electron acceptor for anaerobic respiration of organic matter by chemoheterotrophs, being first reduced to nitrite and then to ammonium. Anaerobic conditions could be present inside stable soil aggregates, for instance (Keiluweit et al., 2017; van den Bergh et al., 2024), so this precondition is not difficult to fulfill even in (otherwise) aerobic soils. The size of nitrate and ammonium pools in the different soils (Figure 4) would suggest this scenario is plausible. However, direct quantification of abundance of carriers of both tested DNRA genes showed significant suppression by the AM fungus (Supplementary Figure 1). Thus, unless the expression of the DNRA genes, which was not measured here, is completely uncoupled from abundance of their microbial carriers, this scenario must be dismissed.

Another possibility would be that the AM fungus did suppress AOB (and comammox *Nitrospira*) independently of a simple competition for substrate, resulting in net accumulation of ammonium in the meshbags, while this pool was less efficiently consumed by the AM fungus than by nitrifiers. Such accumulation could only be transient but since we only measured the N concentrations once, and we did not use isotopic tracers to support this theory, we cannot make conclusions about temporal variation of ammonium or nitrate concentrations as affected by AM fungal hyphae. Yet, this scenario opens new questions, particularly about how the AM fungus may have suppressed the AO microorganisms here, if not through competition for substrate. It is possible (though not demonstrated yet in any case, to the best of our knowledge) that the AM fungus or its associated host plant produced some kind of biological nitrification inhibitor, which eventually reached the root-free soil (possibly transported via AM fungal hyphae), suppressing specifically the AOB and comammox *Nitrospira* (Nardi et al., 2020 and references therein; Teutscherova et al., 2019; Sarr et al., 2021). Our previous research showed that such elusive nitrification inhibitors are unlikely to be produced by the AM fungus alone (Sun et al., 2023), but since we used a C_4_ grass as a host plant (albeit with no reported potential for biological nitrification inhibitor production as yet), the transport of plant metabolites with nitrification inhibition potential via AM fungal networks remains an exciting opportunity for further research.

Still another possibility to explain the “ammonium paradox” would be that the AM fungi prefer nitrate over ammonium, but this is unlikely as it should promote rather than suppress nitrification in consequence (Bukovská et al., 2018). Besides, this contradicts earlier measurements of uptake of different mineral and organic N species by AM fungal hyphae, reporting a clear preference for ammonium over nitrate ions (Hawkins et al., 2000).

Despite the statistically significant general trend of suppression of AOB and comammox *Nitrospira* by AM fungal activity, there was a broad variety of effects across the individual soils, and in some soils the AM fungus apparently promoted rather than suppressed the abundance of AOB or comammox *Nitrospira*, or had no effect on the AO abundance (Figure 3). This is probably an important observation, although we could not rigorously test the variability of the mycorrhizal suppression effect across replicate pots with the same soil due to our experimental design constraints (only using two pots per soil treatment, one inoculated with the AM fungus, and one not). Thus, dedicated experiments including a selection of the soils tested here and proper replication are needed to address such effects rigorously. All that could be done here was to correlate the general trends with soil properties, which boiled them down to soil pH and altitude (the two were strongly and negatively correlated to each other, *p* < 0.001, *R*^2^ = 0.56), texture, and soil organic C, total N, and available P concentrations.

Soil pH was previously described as an important modulator of AO abundance and activity (de Boer et al., 1992; Lin et al., 2021). Here, we also found that pH was positively correlated with AOB response (*p* = 0.018) to AM fungus presence among many parent soil properties (Table 4). The AOA abundance was slightly, but not statistically significantly, suppressed by AM fungus, which is congruent with previous observations (Che et al., 2015; Xiang et al., 2017; Sun et al., 2024). In fact, parent soil properties (NH_4_^+^ and total organic C concentrations, and sandiness) were related to the response ratio of AOA to AM fungus presence (Table 4). Since parent soil NH_4_^+^ was positively correlated with AOA response (*p* = 0.028), the increased soil NH_4_^+^ due to AM fungal presence (Figure 4 and Table 3) could have mitigated the suppression effect of AM fungi on the AOA. In addition, since NH_4_^+^ concentrations in our soils before and after incubation in the pots were relatively low, and it is known that under such conditions the AOA prevail over AOB due to their higher versatility (Wright and Lehtovirta-Morley, 2023), the compounded effect of low mineral N and further exploitation of the ammonium pool by AM fungal hyphae (but see above) could have led to significant suppression of AOB but not AOA, the latter of which are generally more efficient in exploiting low ammonium concentrations (Wright and Lehtovirta-Morley, 2023).

Finally, the loss of microbial taxa (prokaryotes, protists, and AOB) observed here during soil incubation in the pots could be due to the different environmental constraints imposed by the pot experiment. Interestingly, the response of the AOA community in the experimental pots differed from that of the other microbial groups tested, and we observed increase in relative abundance of some AOA taxa when comparing pot experiment to field soils. Field soils receive continuous input of diverse nutrients and/or microbes and are exposed to a greater variation in temperatures and moisture availability across temporal scales than in the glasshouse, which might have promoted particularly adverse condition-tolerant groups such as the AOA.

The prokaryote, protist, and AOB communities indigenous to the different soils and responding to both the incubation in the glasshouse and AM fungus inoculation are likely linked to changes in soil nutrient availability or AM fungal hyphae effects (hyphosphere effect, Faghihinia et al., 2023). The presence of the AM fungus coincided with a decrease of nitrate and an increase of ammonium but caused no major changes to soil pH during pot incubation. In line with the third hypothesis, it is interesting that the AOA community, with regards to the relative abundance of different AOA taxa, was also less responsive to AM fungal inoculation as compared to the other microbial groups (Figure 5 and Table 5). These findings indicate AOA abundances might be influenced by factors which were not measured in this study, which advocates further research into soil AOA ecology.

## 5 Conclusion

By scrutinizing a wide range of agricultural soils collected within the Czech Republic, inoculation with the AM fungus *Rhizophagus irregularis* was in general suppressing the abundance of indigenous AOB and comammox *Nitrospira*. The response ratio of AOB abundance to presence of the AM fungus was significantly correlated with parent soil pH, indicating context dependency of the observed effect and advocating further research in this direction. In contrast, the AM fungus had very little effect on AOA abundance, as it did not influence soil properties (i.e., sand and total organic carbon contents) that clearly determined AOA responsiveness to AM fungus across the different soils. The MRR of comammox *Nitrospira* was governed by a different selection of soil properties compared with both AOB and AOA, indicating their different ecological niche. Contrary to our hypothesis, soil NH_4_^+^ was elevated (and NO_3_^–^ concentration was suppressed) by AM fungal activity despite their assumed competition for NH_4_^+^ with AO microorganisms, and the elevated NH_4_^+^ levels were unlikely due to enhanced DNRA pathway activity. We further found strong effects of the AM fungus on AOB community structure, while such effects were rather limited for the AOA, implying that there are different mechanisms underlying interactions between AM fungi and different AO guilds in soil, which likely go well beyond the nutrient availability and substrate competition. Given the importance of nitrification for soil N cycling, further research into AM fungi-AO interactions is warranted, particularly in soils under various development or management legacies. A future focus on temporal dynamics of such effects is strongly advocated.

## Data Availability

All primary data are provided in Supplementary Data accompanying this article. The raw sequencing data generated in this study were deposited in the Sequence Read Archive of the NCBI under the accession number PRJNA1085112.

## Footnotes

1
www.ncbi.nlm.nih.gov

2
www.arb-silva.de

3
www.pr2-database.org
