# Supplementary material for: Arbuscular mycorrhiza suppresses microbial abundance, and particularly that of ammonia oxidizing bacteria, in agricultural soils
Source: Front Microbiol. 2025 Nov 18;16:1671859. doi: 10.3389/fmicb.2025.1671859 (PMC12669096; doi:10.3389/fmicb.2025.1671859)
Supplement: Supplementary file 1 [file Data_Sheet_1.pdf]

Supplementary Figures

Arbuscular mycorrhiza suppresses microbial abundance, and particularly that of ammonia oxidizing bacteria, in agricultural soils

Daquan Sun[1], Petr Šmilauer[2], Petra Pjevac[3], Martin Rozmoš[1], Sándor T. Forczek[1], Michala Kotianová[1], Hana Hršelová[1], Petra Bukovská[1], Jan Jansa[1*]

[1]Laboratory of Fungal Biology, Institute of Microbiology, Czech Academy of Sciences, Vídeňská 1083, 14200 Praha 4, Czech Republic

[2]Faculty of Science, University of South Bohemia in České Budějovice, Branišovská 1645/31a 37005 České Budějovice, Czech Republic

[3]Joint Microbiome Facility/Division of Microbial Ecology (DOME), Centre for Microbiology and Environmental Systems Science, University of Vienna, Djerassiplatz 1, 1030 Vienna, Austria

*** Correspondence:**
Jan Jansa
jansa@biomed.cas.cz

tel +420736404166

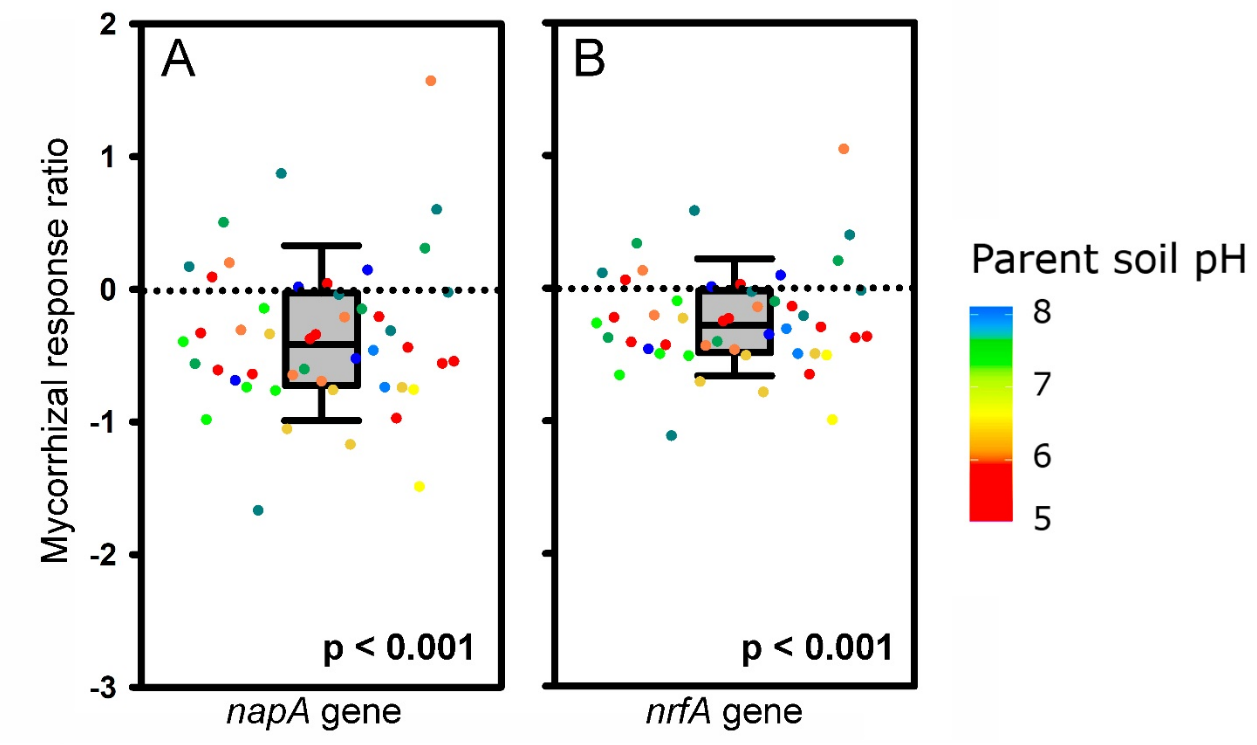

Supplementary Figure S1. Mycorrhizal response ratio (MRR; n=48) of abundance of genes in the meshbags, which are involved in dissimilatory nitrate reduction to ammonium pathway: (A) nitrate reductase (*napA*) and (B) nitrite reductase (*nrfA*). MRRs are displayed as medians, 25% and 75% percentiles (boxes) and 5% and 95% percentiles (error bars) of MRRs calculated separately for each soil (dots), using individual values measured in meshbags placed in mycorrhizal (AM) and non-mycorrhizal (NM) pots. The p-values refer to non-parametric one-sample Wilcoxon signed-rank test scrutinizing differences of sample medians from zero. Dot colors stand for pH values of the parent soils.

Prokaryotes (16S gene)

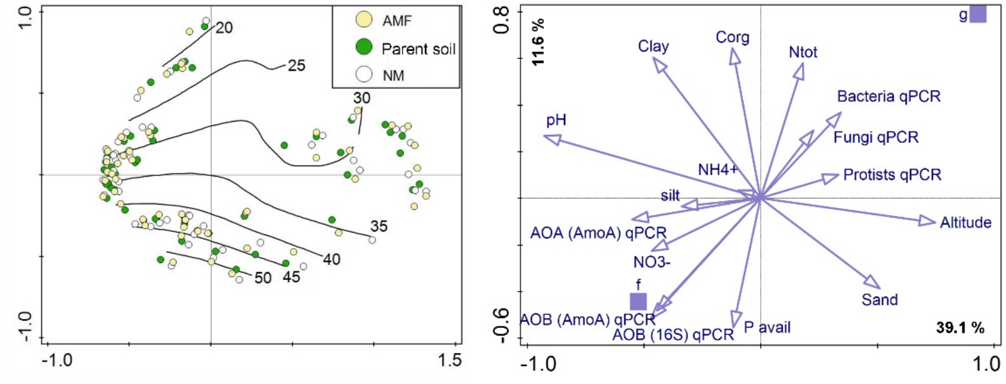

Protists (18S gene)

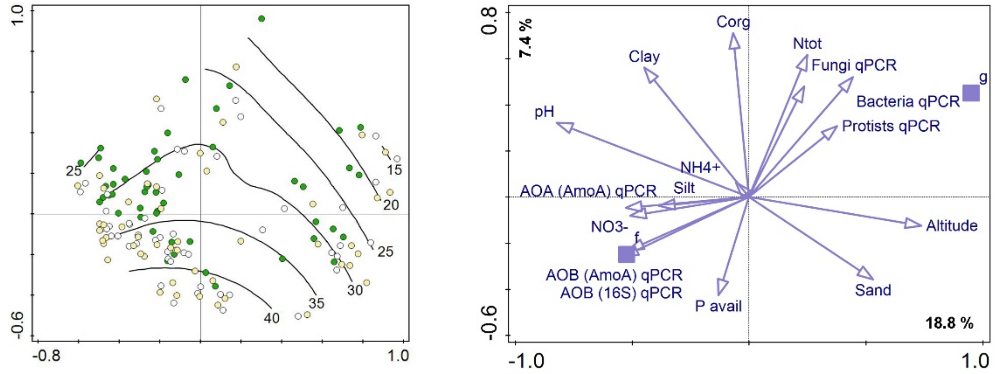

Ammonia oxidizing bacteria (*amoA* gene)

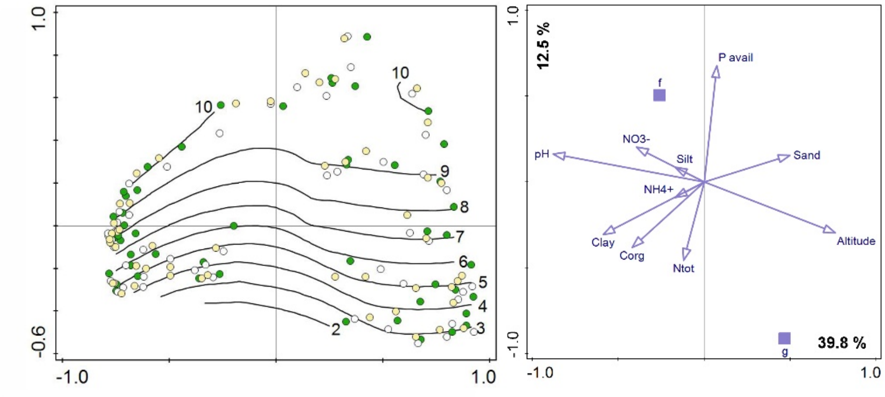

Ammonia oxidizing archaea (*amoA* gene)

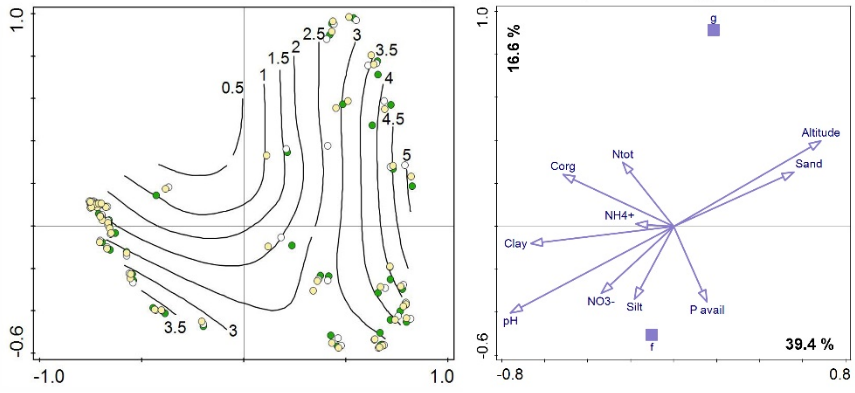

Supplementary Figure S2. Principal component analyses exploring the relationship between microbial community profiles in the different soils (both field soils before incubation in the pots termed “Parent soil” are included and post-incubation in the meshbags, either in the non-mycorrhizal (NM) setup or with arbuscular mycorrhizal fungus (AMF) inoculated to the plants) and physico-chemical and biological properties of the soils. Contour plots (left) showing changes in community diversity (richness) across the samples in the ordination space. Individual samples are projected to the ordination space. Predictor scatterplots are shown to the right, indicating projection of the soil physico-chemical predictors along with quantitative descriptors of microbial guilds (only for prokaryotes and protists) onto the first two axes of the ordination space. Centroids for the field (f) and grassland (g) samples are shown. Bold numbers indicate the fraction of dataset variability explained by the first and second axes.

Prokaryotes (16S) - 741 OTUs

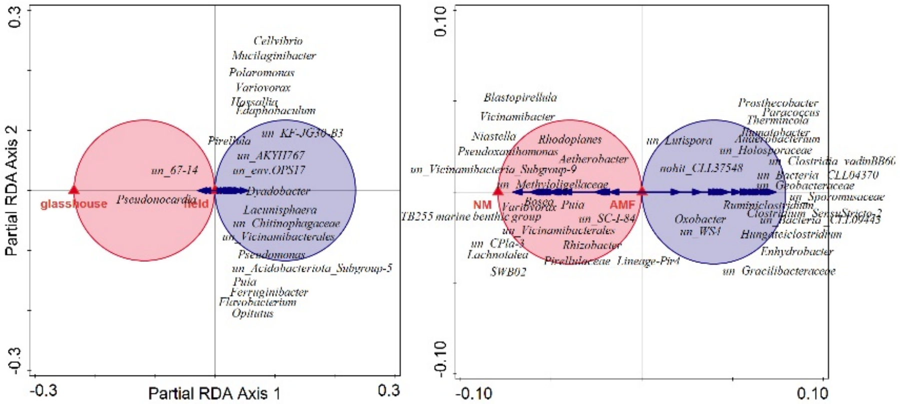

Protists (18S) - 1103/1081 OTUs

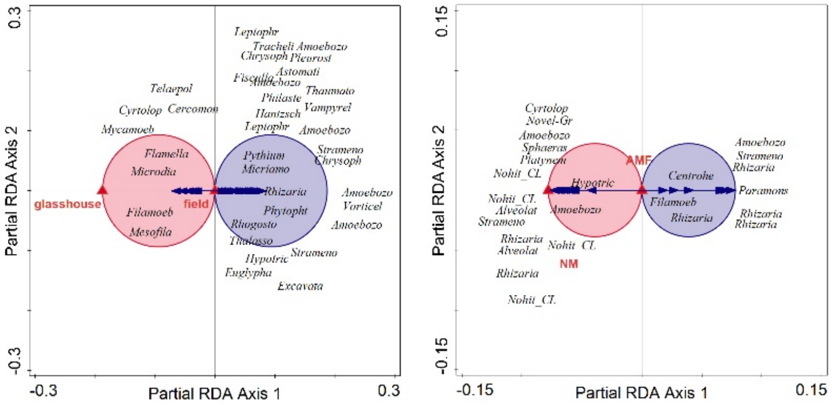

Ammonia oxidizing bacteria (*amoA* gene) - 644/641 OTUs

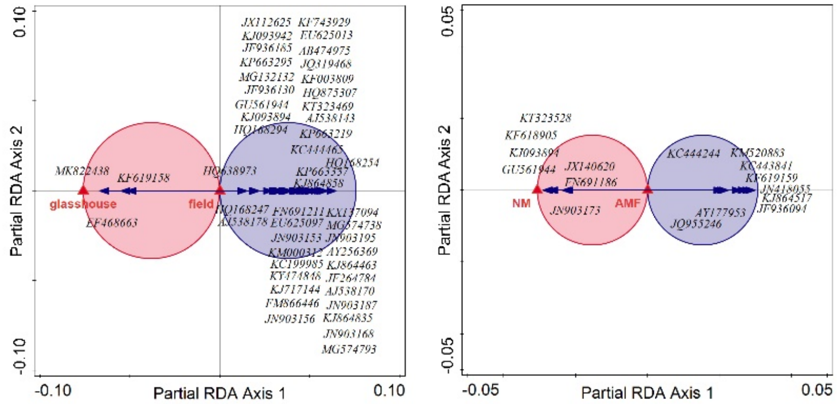

Ammonia oxidizing archaea (*amoA* gene) - 387 OTUs

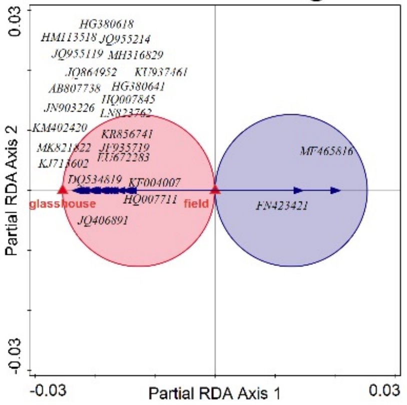

partial redundancy analysis p > 0.05

Supplementary Figure S3. T-value biplots following partial redundancy analysis (RDA) and detailing microbial taxa which were significantly affected by incubation of experimental soils in pots (contrast field × glasshouse, left) or presence vs. absence of the arbuscular mycorrhizal fungal inoculum in pots (inoculated with *Rhizophagus*, AMF, or non-mycorrhizal, NM treatment, right). Taxa names are truncated or replaced with GenBank accession numbers (closest relatives) for better overview.
